# Reported food-related symptoms and food allergen sensitization in a selected adult population in Hyderabad, India: A hospital-based survey

**DOI:** 10.1016/j.jacig.2023.100204

**Published:** 2023-12-23

**Authors:** Priyanka Dadha, Sai Nimmagadda, Carina Venter, Ruchi Gupta, Putcha Uday Kumar, Christopher M. Warren

**Affiliations:** a Center for Food Allergy & Asthma Research (CFAAR), Institute for Public Health and Medicine, Northwestern University Feinberg School of Medicine, Chicago, Ill; b Ann & Robert H. Lurie Children’s Hospital of Chicago, Chicago, Ill; c Department of Pediatrics–Allergy/Immunology, CFAAR, Chicago, Ill; d Division of Pathology & Microbiology, Indian Council of Medical Research–National Institute of Nutrition, Ministry of Health and Family Welfare, Government of India, Hyderabad, Telangana, India

**Keywords:** Atopic disease, food-related atopic symptoms, skin prick test, food specific IgE, South India

## Abstract

**Background:**

Research on reported food-related triggers of atopic disease in South Asian adults is lacking despite the region’s large population and the global significance of allergic diseases.

**Objectives:**

The study aimed to identify prevalent local food items and assess allergic sensitization rates to potential trigger foods for atopic diseases via skin prick and specific IgE testing.

**Methods:**

The study began with a pilot survey of 100 subjects recruited from 4 hospitals in Hyderabad, India, focusing on foods perceived to relate to asthma, allergic rhinitis, atopic dermatitis, urticaria, and gastrointestinal allergic symptoms. A subsequent main study evaluated 2010 participants, 1754 of whom were diagnosed with an aforementioned atopic disease and who reported allergic symptoms related to any of 77 foods identified in the pilot study. Ultimately 1622 patients who consented to skin prick and specific IgE testing and who reported at least 1 food item triggering allergic diseases were included in the final analysis.

**Results:**

Among 1622 patients (average age, 42.6 ± 12.9 years; 55.5% male), asthma was the most commonly diagnosed atopic disease (26.4%), with itching and rash being frequently reported symptoms (22.7%). Notably, 94.9% of patients had total serum IgE > 144 kU/L. Chickpea, cabbage, eggplant, walnut, cumin, and betel leaf were the most commonly reported trigger foods.

**Conclusion:**

In this sample of South Indian adults diagnosed with allergic disease, reported food triggers were most commonly local dietary staples, while reported reactions to priority allergens like peanut and sesame were conspicuously absent. Observed concordance between patient-reported food triggers and sensitization to reported food triggers was low, highlighting the need for improved clinical evaluation of suspected triggers.

Atopic diseases (food allergy, atopic dermatitis, asthma, and allergic rhinitis) affect about 20% of the global population.^1^ The coexistence of these diseases^2^ may increase the risk of severe food-induced anaphylactic reactions.^3^ New-onset food allergies are also being observed in adults,^4^ highlighting the need to understand the relationship between food intake and atopic disease outcomes. Symptoms of food-related atopic diseases can develop rapidly, involving respiratory, cutaneous, gastrointestinal, and cardiovascular systems.^5^ Understanding the association between food consumption and atopic disease symptoms is crucial for public health, considering various factors like preparation methods, timing of food exposure, and environmental cofactors.^6^ The reasons for regional variations are not fully understood.

Studies from Asia show a wide variety of food items perceived as common triggers for atopic symptoms that are different from those in the United States, Europe, and other countries.^7^, ^8^, ^9^, ^10^, ^11^, ^12^ Food-related atopic symptom trends also vary across ethnic groups, with Asian Indian adults having a unique diet. Studies^13^, ^14^, ^15^, ^16^, ^17^, ^18^ conducted in India identified legumes, brinjal, cucumber, ladyfinger, papaya, pomegranate, and banana as major triggers. However, these studies had limitations like self-report,^13^^,^^14^ specific disease conditions,^15^^,^^16^ small sample size,^16^^,^^17^ and international reporting,^18^ and were not representative of Indian dietary patterns. Reaction symptomatology varies among individuals and may lead to anaphylaxis. In India, there is a lack of epidemiologic data on food-induced anaphylaxis, possibly as a result of inadequate case recognition and reporting within the health care system.^19^ Incidence rates may be historically low or underreported.

A clinical diagnosis of suspected food-related atopic disease is crucial to distinguish them from other non–immune-mediated adverse reactions.^5^ Obtaining a proper diagnosis helps limit unnecessary dietary restrictions and potential nutritional deficiencies, easing the burden on patients, caregivers, and families.^20^^,^^21^ Current clinical practice parameters recommend using skin prick test (SPT) and measuring total serum IgE and specific IgE (sIgE) for empirical evaluation. The reference standard for diagnosis is the double-blind, placebo-controlled food challenge; however, this is expensive and is not routinely conducted in research settings.

Patient self-report tends to overestimate atopic outcomes.^22^ However, the extent of disparity between perceived and confirmed food-related atopic symptoms in India is unclear. Further studies are necessary to accurately validate patient reports.

The study aimed to identify perceived allergic food triggers and estimate rates of allergic sensitization to these trigger foods in Hyderabad, India. Patients with perceived symptoms to 77 reported trigger foods underwent evaluation using SPT, serum total IgE, and sIgE to confirm sensitization.

## Methods

### Study site

The study was conducted at Indian Council of Medical Research (ICMR)-National Institute of Nutrition (NIN), Hyderabad, India, and patient enrollment was conducted at 3 governmental and 1 private hospital in Hyderabad. Patients aged 18 to 60 with clinically diagnosed atopic diseases (asthma, allergic rhinitis, atopic dermatitis, urticaria, and gastrointestinal-induced allergic symptoms) were included. The study period was from July 2016 to June 2018.

The study had specific exclusion criteria, including pregnancy or lactation, malignancy, and severe systemic diseases. Ethical approval was obtained from the ethics committee of ICMR-NIN and the private and government hospitals in Hyderabad. Informed consent was obtained from all participants before their participation in the study.

### Definitions

Perceived food-related atopic disease was defined as patient-reported symptoms associated with any of the listed food items linked to their atopic disease diagnosis (asthma, allergic rhinitis, atopic dermatitis, urticaria, and gastrointestinal-induced allergic symptoms). Probable food-related atopic disease was defined as the combination of reported symptoms along with sensitization to the food, where food sensitization was determined by sIgE > 0.35 IU/mL.

Considering a cumulative prevalence of atopic diseases at 5% to 8% with a 95% confidence interval and a relative precision of 20%, the study was initially designed to enroll a minimum of 1825 participants. However, the study eventually included a total of 2010 adult participants (aged 18-60 years).

### Study design

This observational, cross-sectional study aimed to identify cases of perceived and probable food-related allergic symptoms based on SPT results and a convincing IgE-mediated report.

The study consisted of a pilot survey (Annexure I), followed by a screening survey (Annexure IIa) and a main survey (Annexure IIb). The surveys were developed by the study team and pretested with 50 participants to ensure clarity, relevance, validity, and reliability of the questions and response options. Feedback from the pretest was incorporated into the final questionnaire. Face validity was assessed by 2 experts, and test–retest reliability showed excellent correlation (*r* > 0.75) on 20 questionnaires before data collection commenced.

**Fig 1:**
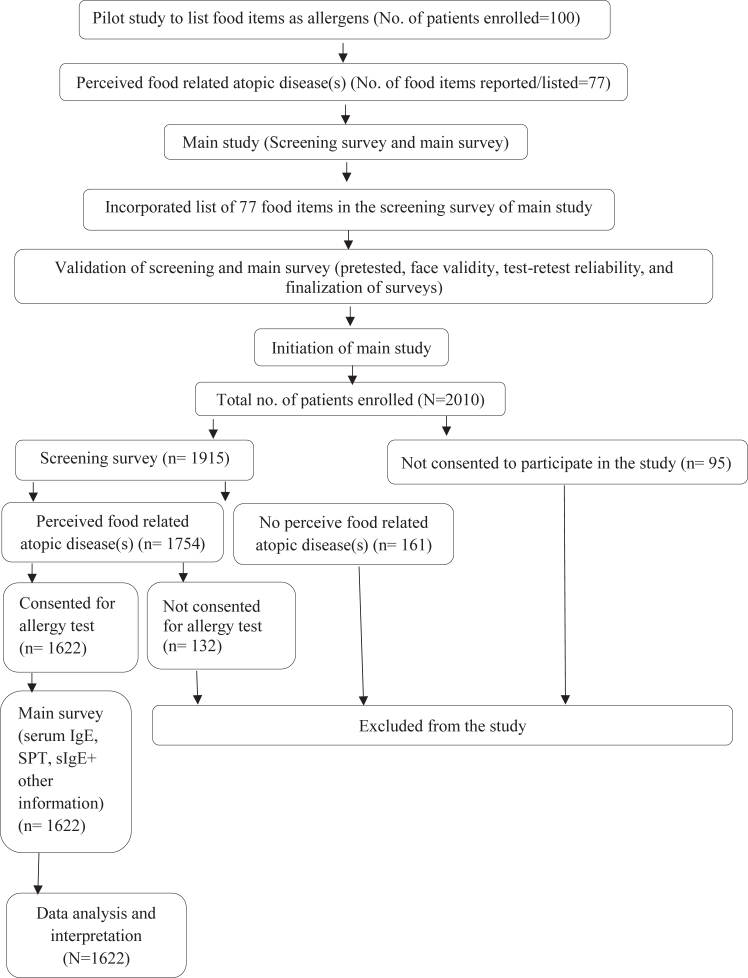
Methodology flowchart.

The detailed methodology of the study is shown in Fig 1, and patient characteristics are listed in Table I.

**Table I:** Demographic profile of adult (18-60 years old) participants enrolled onto main study

Characteristic	Total enrolled (N = 1915)		Total perceived food-related atopic disease (n = 1754)	
	With perceived food-related atopic disease (n = 1754)	Without perceived food-related atopic disease (n = 161)	Consented to testing (n = 1622)	Did not consent to testing (n = 132)
Sex				
Male	972 (55.4)	88 (54.7)	900 (55.5)	72 (54.5)
Female	782 (44.6)	73 (45.3)	722 (44.5)	60 (45.5)
Age group				
18-25 years	253 (14.4)	27 (16.8)	233 (14.4)	20 (15.2)
25-40 years	491 (28.0)	53 (32.9)	464 (28.6)	35 (26.5)
40-60 years	1010 (57.6)	81 (50.3)	925 (57.0)	77 (58.3)
Mean age (years)	42.6 ± 12.8	40.6 ± 12.9	42.6 ± 12.9	42.8 ± 13.0
SES				
Upper	525 (29.9)	44 (27.3)	483 (29.8)	42 (31.8)
Upper middle	343 (19.6)	26 (16.1)	319 (19.7)	24 (18.2)
Lower middle	302 (17.2)	34 (21.1)	280 (17.3)	22 (16.7)
Upper lower	338 (19.3)	30 (18.6)	312 (19.2)	26 (19.7)
Lower	246 (14.0)	27 (16.8)	228 (14.1)	18 (13.6)
Smoking				
Never	901 (51.4)	83 (51.6)	833 (51.4)	68 (51.5)
Ex-smoker	741 (42.2)	66 (41.0)	685 (42.2)	56 (42.4)
Current	112 (6.4)	12 (7.5)	104 (6.4)	8 (6.1)
Diagnosis				
A	466 (26.6)	35 (21.7)	429 (26.4)	37 (28.0)
AR	388 (22.1)	35 (21.7)	358 (22.1)	30 (22.7)
A + AR	269 (15.3)	32 (19.9)	253 (15.6)	16 (12.1)
A + U	345 (19.7)	35 (21.7)	318 (19.6)	27 (20.5)
A + AR + U	238 (13.6)	21 (13.0)	220 (13.6)	18 (13.6)
AR + U	48 (2.7)	3 (1.9)	44 (2.7)	4 (3.0)
Symptoms				
Itching and rash	389 (22.2)	NA	368 (22.7)	41 (31.1)
Breathlessness	377 (21.50	NA	349 (21.5)	38 (28.8)
Runny, stuffy nose	356 (20.3)	NA	349 (21.5)	37 (28.0)
Diarrhea or vomiting (not food poisoning)	262 (14.9)	NA	241 (14.9)	21 (15.9)
Headache	140 (8.0)	NA	122 (13.7)	8 (6.1)
Fainting or dizziness	130 (7.4)	NA	113 (7.0)	7 (5.3)
Duration of symptoms				
1-5 years	429 (24.5)	NA	397 (24.5)	32 (24.2)
5-10 years	408 (23.3)	NA	378 (23.3)	30 (22.7)
10-15 years	313 (17.8)	NA	294 (18.1)	19 (14.4)
15-20 years	308 (17.6)	NA	283 (17.4)	25 (18.9)
20-25 years	259 (14.8)	NA	283 (17.4)	23 (17.4)
>25 years	37 (2.1)	NA	34 (2.1)	3 (2.3)
Family history				
Yes	1235 (70.4)	114 (70.8)	1140 (70.3)	95 (72.0)
No	519 (29.6)	47 (29.2)	482 (29.7)	37 (28.0)
Serum IgE levels				
Atopic (>144 kU/L)	1666 (95.0)	152 (94.4)	1539 (94.9)	127 (96.2)
Mean ± SD	386.0 ± 180.8	378.3 ± 180.4	385.4 ± 181.3	392.5 ± 182.0
Nonatopic (1.5-144 kU/L)	88 (5.0)	9 (5.6)	83 (5.1)	5 (3.8)
Mean ± SD	134.8 ± 8.0	133.6 ± 7.3	134.7 ± 7.9	137.3 ± 10.3

Data are presented as nos. (%) unless otherwise indicated.

*A,* Asthma; *AR,* allergic rhinitis; *NA,* not applicable; *U,* urticaria.

#### Pilot study

A pilot study of 100 subjects was conducted for 30 days, during which patients listed food items perceived to be related to their atopic diseases diagnosed by specialists (pulmonologist, dermatologist, ear, nose, and throat specialist, or gastroenterologist). The purpose was to develop a list of perceived food items associated with allergic symptoms and to order commercial extracts for SPT and sIgE testing in the main study from ALICIT India Private Ltd.

#### Main study

The screening survey enrolled a total of 2010 patients based on inclusion and exclusion criteria. Of these, 95 patients did not consent to participate, leaving 1915 patients who completed the screening survey.

### Main study surveys

Data collection for the main study comprised 2 questionnaires: the screening survey and the main survey.

#### Screening survey

The screening survey, which was conducted with 1915 subjects, included the list of 77 food items (listed in the Online Repository at www.jaci-global.org) from the pilot study and collected demographic information. In order to progress through the study, the participant had to positively reply to 2 questions: first, was the diagnosis made by the practitioner? (ie, which of the abovementioned atopic diseases has been diagnosed by the practitioner?); and second, have you ever had these abovementioned atopic diseases caused by eating any of the following foods? (with 77 food items listed). The atopic disease or diseases considered were the same as during the pilot study and screening.

Of 1915 patients, 161 did not perceive their diagnosed atopic disease related to any 77 listed food items and were excluded from further interrogation. Thus, 1754 participants progressed to the main survey.

#### Main survey

A total of 1754 patients with reported allergic diagnoses and food-related symptoms were included in the main survey, which collected demographic information and symptoms after eating the reported food items as well as symptom duration. Biochemical and serologic tests (SPT, total serum IgE, and sIgE) were conducted for each participant. Of the 1754 patients who entered the main survey after screening, 132 patients did not consent to sensitization or allergy testing. Therefore, 1622 patients were included in the final analyses.

### Sensitization testing

SPT and sIgE testing were conducted for all 77 listed food items in the remaining 1622 patients. SPTs were performed using commercially available reagents from ALICIT India Private Ltd. A positive SPT reaction to histamine, along with a negative reaction to the negative control, served to validate the accuracy of the SPT for the individual being evaluated. Patients were classified as SPT positive for a specific food allergen if they exhibited a positive reaction, defined as a wheal diameter of ≥3 mm, while also showing a negative reaction to an uncoated lancet. SPT was scheduled after taking a detailed history and was based on patient availability. Corticosteroid and antihistamine receipt were prohibited 1 week before SPT and blood collection to avoid masking severe symptoms. Total serum IgE levels were estimated by ELISA (Adaltis), with normal range defined as between 1.5 and 144 kU/L.^23^

SPTs and blood draws were conducted by a trained team in their respective hospitals under the lead author’s supervision. Serologic investigations and statistical analyses were performed at ICMR-NIN in Hyderabad. Serum samples were shipped to Metropolis Healthcare Ltd, Mumbai, and ELISA tests were performed for semi-quantitative sIgE estimations; sIgE > 0.35 IU/mL was considered positive.

### Data analysis

Data were analyzed by SPSS v22 software (IBM). Categorical data are represented by frequencies and percentages, and continuous data are represented as means ± SDs.

## Results

The main study enrolled 1915 patients, and 1622 patients (mean age, 42.6 ± 12.9 years) were included in the final analyses. More male patients (55.5%) perceived food-related symptoms than female patients (44.5%). Patients were distributed across different age groups: 14.4% were aged 18-25 years, 28.6% were aged 25-40 years, and 57.0% were aged 40-60 years. According to the Kuppuswamy scale,^24^ patients were classified into different socioeconomic strata: 29.8% upper, 19.7% upper middle, 17.3% lower middle, 19.2% upper lower, and 14.1% lower socioeconomic status (SES).

Among the 1622 patients with perceived food-related symptoms, 51.4% were never smokers, 42.2% were ex-smokers, and 6.4% were current smokers. The most common atopic conditions were asthma alone (26.4%) and allergic rhinitis alone (22.1%), followed by combinations like asthma–urticaria (19.6%), asthma–allergic rhinitis (15.6%), asthma–allergic rhinitis–urticaria (13.6%), and allergic rhinitis–urticaria (2.7%). Reported symptoms included breathlessness, runny/stuffy nose, itching/rash, diarrhea or vomiting (not food poisoning), headache, and fainting or dizziness. Most patients experienced symptoms for 1 to 5 years (24.5%) and 5 to 10 years (23.3%).

Among the patients with perceived food-related atopic disease, a higher proportion had a family history (70.3%) compared to those with no family history (29.7%), based on self-report only. A total of 1539 (94.9%) of 1622 patients with perceived symptoms were atopic, with a mean total serum IgE level of 385.4 ± 181.3 kU/L, which exceeded the normal range (1.5-144 kU/L).

SPT and sIgE were assessed for 1622 of 1915 participants. Among the 77 listed food items, concurrence among perception, SPT, and sIgE was found for chickpea, cabbage, eggplant, walnut, cumin, and betel leaf (Table II). Detailed data for all 77 food items are available in the Online Repository.

**Table II:** Food items that showed alignment between perceived food-related atopic disease, positive SPT results, and food-specific IgE levels in 1622 subjects

Food item (local telugu name)	Perceived food-related atopic disease	SPT positive	sIgE positive
Cereals and pulses			
Chickpea (chingalu)	524 (28.8)	115 (6.3)	21 (1.2)
Vegetables			
Cabbage (cabbage)	766 (42.1)	39 (2.1)	1 (0.1)
Eggplant (vankay)	448 (24.6)	27 (1.5)	4 (0.2)
Nuts			
Walnut (bikki pica)	224 (12.3)	105 (5.8)	20 (1.1)
Other commonly consumed food items			
Cumin (jeelakara)	232 (12.7)	107 (5.9)	25 (1.4)
Betel leaf (tamala paku)	152 (8.4)	24 (1.3)	2 (0.1)

Data are presented as nos. (%). SPT positivity was defined as wheal ≥3 mm in diameter; sIgE positivity, >0.35 IU/mL.

Of the 77 listed food items, alignment (perception, SPT, and sIgE) was found for 3 food items (chickpea, walnut, and cumin) across all age groups (Table III). It can be interpreted that 3 of 77 food items had a low level of alignment between the 3 parameters. Detailed data on alignment by age group for all 77 food items are available in the Online Repository.

**Table III:** Food items that showed alignment between perceived food-related atopic disease, positive SPT result, and sIgE by age group

Food item (local telugu name)	Age group (N = 1622)								
	18-25 years (n = 233)			25-40 years (n = 464)			40-60 years (n = 925)		
	Perceived food-related symptoms	SPT positive	sIgE positive	Perceived food-related symptoms	SPT positive	sIgE positive	Perceived food-related symptoms	SPT positive	sIgE positive
Cereals and pulses									
Chickpea (chingalu)	53 (22.7)	7 (3.0)	3 (1.3)	124 (26.7)	42 (9.1)	8 (1.7)	314 (33.9)	60 (6.5)	8 (0.9)
Nuts									
Walnut (bikki pica)	50 (21.5)	9 (3.9)	2 (0.9)	60 (12.9)	39 (8.4)	10 (2.2)	97 (10.5)	52 (5.6)	5 (0.5)
Other commonly consumed food items									
Cumin (jeelakara)	12 (5.2)	12 (5.2)	1 (0.4)	70 (15.1)	36 (7.8)	8 (1.7)	127 (13.7)	55 (5.9)	14 (1.5)

Data are presented as nos. (%). SPT positivity was defined as wheal ≥3 mm in diameter; sIgE positivity, >0.35 IU/mL.

## Discussion

To our knowledge, our study is the first to comprehensively characterize the burden of food-related atopic disease symptoms in the Indian population. Findings suggest that South Indian patients with a current diagnosis of atopic disease who associate their symptoms with certain food items are not clinically sensitized to most of these suspected food triggers. However, most patients had elevated total serum IgE levels. Sensitization was confirmed for common allergens like chickpea, eggplant, walnut, coconut, prawn, mango, and local foods like betel leaf. A similar study^18^ conducted in Mysore and Bangalore, Karnataka (India), observed a high level of sensitization (26.5%) to most foods (based on 24 priority EuroPrevall food items) consumed by the general population. However, the prevalence of probable food-related atopic symptoms based on self-report and sIgE was only 1.2%, mainly associated with cow’s milk (0.5%) and apple (0.5%). Sensitization was predominantly seen in individuals with total IgE levels above the median.

Asia is the most populous continent, and South Asian countries, including India, have diverse ethnic groups, cultures, languages, traditions, religions, and dietary patterns. India has 2 distinct populations with variations in diet and climate across regions. Northern India’s staple diet is wheat, with spicier and heavier foods that take more time to digest because they may contain more ghee, butter, or fat; the south’s staple is rice, with lighter and more vegetarian dishes as well as more consumption of fermented food items. Coconut is native to South India, and eating on banana leaves is a traditional practice.^25^^,^^26^ Hyderabad, the capital city of Telangana, is a multicultural hub with a significant migrant population.^27^ As of 2022, Hyderabad’s population is estimated to be 10.6 million, with a sex ratio of 945 female per 1000 male inhabitants.^28^

The study aimed to investigate sensitization to perceived food triggers among patients with asthma, allergic rhinitis, atopic dermatitis, urticaria, and gastrointestinal-induced allergic symptoms in Hyderabad, India, where information on regional food-related atopic comorbidities is limited.

Previous studies^13^^,^^15^^,^^18^^,^^29^^,^^30^ conducted in different zones of India reported associations between various food items and allergic symptoms, but they were based on self-report without confirmation. Some food items, like cow’s milk and hen’s egg, were commonly reported allergens across all regions. In the present study in Hyderabad, 91.6% of participating patients perceived at least 1 commonly consumed food item to be associated with their diagnosed atopic diseases, indicating a high level of self-imposed dietary restriction that may lead to nutritional deficiencies.

The present study found that most patients were male, were 40 to 60 years old, were from upper SES, never smoked, and had a family history of atopic disease. Commonly reported symptoms were breathlessness, itching, urticaria, and runny/stuffy nose, with diagnoses of asthma, allergic rhinitis, and atopic dermatitis.

Various global studies have reported demographic profiles similar to and different from the 1622 patients in the present study with perceived food-related atopic disease. For instance, a European study^31^ (N = 2185; subjects aged 20-54 years) found a higher prevalence of adult food allergy in younger age (odds ratio [OR] = 0.97) and male sex (OR = 1.39), with major symptoms being allergic asthma (14.8%), allergic rhinitis (50%), and atopic dermatitis (18%). Another study^30^ showed higher perceived food-related atopic symptoms in women (20.6%) compared to men (15.2%), with a decline in prevalence with age (*P* < .01). Risk factors for food-related symptoms included allergic diseases, atopic family history, higher education level, and family income (*P* < .0001). A similar study^32^ (N = 3433) revealed that perceived food-related atopic symptoms were greater in women (20.6%) compared to men (15.2%) and declined with age (*P* < .01), as the lowest prevalence was found among those aged >60 years (6.7%). Another study^33^ found that younger age (OR = 0.99) and female sex (OR = 1.87) were associated with a higher risk of food-related atopic symptoms (*P* < .001). The most reported symptoms like breathlessness, itching, urticaria, and runny/stuffy nose cannot be solely attributed to food, as there may be overlap with pollen allergies and cross-reactivity.^34^ Pollen allergies are a growing concern worldwide,^35^ and they are associated with food-allergic disorders due to cross-reactivity. Changes in pollen abundance and distribution may indirectly affect these conditions.^36^ Studies have shown that 30% to 60% of food allergies are associated with pollen allergies,^37^ suggesting that the reported symptoms related to food items could be influenced by rising pollen productivity and cross-reactivity.^38^

Sparse concordance was observed between self-reported perceived food-related symptoms and findings from the diagnostic tools used in the present study. Similar results were reported in a United States–based study^39^ (N = 78,851), where participants reporting sesame allergy (0.49%) had a low rate of confirmed diagnosis based on IgE-mediated reactions (0.23%). Other studies in the United States^40^^,^^41^ and the United Kingdom^42^ have also shown discrepancies between perceived triggers and confirmed diagnoses based on SPT and sIgE testing. Participants often perceived their symptoms to be based on their own assumptions (n = 6620; 69.8%) rather than a clinician’s diagnosis (n = 843; 13.2%).^33^

There was some degree of concurrence to only 6 (chickpea, cabbage, eggplant, walnut, cumin, and betel leaf) of the 77 food items based on the 3 evaluation tools. Chickpea, the second most consumed food in India, displayed 4.2% allergy prevalence with potential IgE-mediated reactions.^43^ Cabbage, a staple vegetable in Indian cuisine, contains allergenic proteins and chitinase allergens with possible cross-reactivity with other lipid transfer protein–containing plants such as cauliflower, broccoli, mustard, rape, and turnip.^44^^,^^45^ Eggplant, widely consumed in India, is a commonly reported food allergy trigger with correspondingly high rates of sensitization.^13^ It contains allergens like A-like nsLTP^46^ and Sola m PPO4.^47^ Walnut, a common tree nut allergen,^48^ showed correlations with birch pollen allergens (Jug r 5; *P* = .92)^34^ and possible cross-reactivity with pecan (Jug r 7; *P* = .92).^49^^,^^50^ Cumin exhibited cross-reactivity with peanut and almond^51^ and was linked to food-induced anaphylaxis.^52^ Betel chewing, a prevalent activity in Hyderabad, was associated with chronic urticaria cases.^53^ Betel plant inflorescence^54^ and eugenol^55^ cause allergic contact dermatitis. Perceived coffee-related atopic diseases had positive SPT reactions but no positive sIgE results. Variations in antibody detection between tests may be due to the absence of IgE antibodies in serum but their presence in tissue mast cells.^56^ Studies in Iraq^57^ and India^29^ reported positive SPT reactions for coffee (25.7% and 4.88% respectively), suggesting cross-reactivity and genetic factors contributing to coffee allergies.^58^ Mechanisms are not fully understood. A plausible explanation for the high SPT positivity to coffee in India may be due to the cultivation of coffee plants, leading to sensitization through exposure to coffee plant pollen or occupational factors.^59^ Similarly, in the case of wheat SPT positivity, a positive SPT for wheat may be observed in individuals with hay fever, but this does not necessarily imply that these individuals cannot consume wheat-based food products like bread, pasta, or porridge without experiencing an allergic reaction.^60^

Our study highlights the significant impact of age on individual perceptions and probable food-related allergic symptoms. There was limited agreement across all age groups in adults for 3 food items (chickpea, walnut, and cumin), with a higher prevalence of probable food-related atopic diseases in the 25-to-40 age group. Other research studies have also emphasized the role of age in triggering or exacerbating food-related atopic diseases. For instance, a study in Canada^61^ found that peanut (OR = 2.24), tree nut (OR = 1.73), and sesame (OR = 5.63) allergies were more common in younger individuals, while fish (OR = 0.17) and shellfish (OR = 0.29) allergies were less common in children. Additionally, factors such as place of residence, SES, and birthplace may also influence the development of food-related atopic diseases.

Published literature from the Indian subcontinent has identified major allergens like mustard,^62^ tomato,^63^ eggplant,^64^ and chickpea,^43^ with their correlations among participants from different age groups being significant. For instance, a study in Mysore with 741 participants (aged 5-60 years) reported adverse reactions to eggplant ingestion in 9.2% of participants. Among those who underwent evaluation, 0.8% tested positive for eggplant allergen sIgE, with most sensitized individuals in the 16-to-45 age group, and female subjects being twice as likely to be sensitized as male subjects.^64^

The present study did not find peanut or sesame to be reported as trigger or to exacerbate any of the diagnosed atopic diseases. While peanuts and sesame are common food allergens,^39^^,^^65^ they are not major sources of allergies for children in Asia,^6^^,^^66^ including India. Early introduction of peanuts during infancy is suggested to prevent peanut allergy.^67^ The burden of sesame allergy is increasing, and India is one of the largest producers and consumers of sesame seeds globally.^68^ There is a significant risk of life-threatening anaphylaxis associated with sesame allergy.^39^ Sesame oil is extensively used in cooking and in traditional medicine for pain relief. A French study^69^ revealed the presence of masked allergens in sesame seed oil, indicating potential allergic reactions; histamine release assays conducted on 5 subjects showed positive results in 3 cases with sesame seed flour and 1 case with sesame seed oil. The absence of reporting for peanut and sesame in the study might be due to their widespread consumption in various forms from fetal development to old age in Indian dietary patterns. However, specific testing for peanut and sesame was not conducted in the study, so the prevalence of peanut sensitization cannot be concluded.

Our study had several limitations, including the lack of SPTs and sIgE testing for patients without perceived food-related atopic disease and the absence of double-blind, placebo-controlled food challenges to confirm clinical reactivity to food triggers. The study sample was recruited from regional hospitals, which may not be fully representative of the general population. Three quarters of the hospitals were government funded, potentially introducing differences in patient demographics and lifestyle compared to private hospitals. The extensive list of food items in the survey may have led to overreporting or overestimation of food triggers as a result of biased responses.

Future studies should focus on longitudinal retrospective and prospective cohort studies with case and control groups to identify and understand the prevalence of food-related atopic diseases in India and to enhance disease surveillance efforts. Collaborative international studies exploring the relationship between food-related atopic diseases, dietary patterns, lifestyle, gut microbiota, and fatty acids should be pursued. It may be worthwhile to explore whether a positive SPT for certain food items, such as coffee or wheat, indicates sensitization to these items at a botanical level rather than as dietary substances, and whether this form of sensitization carries clinical implications. Additionally, sensitivity and specificity studies, along with component-resolved diagnosis on food-related atopic diseases, can provide valuable insights into true food allergens, differentiating primary sensitization from cross-reactivity and predicting reaction severity. Establishing a comprehensive database on region-specific true food allergens across India is essential. Moreover, conducting studies to assess patients’ nutritional status, steroid intake, and their association with body mass index can offer valuable insights into these conditions.

In conclusion, this study identified 77 food items perceived to trigger and/or exacerbate atopic diseases, but alignment between reported and probable food-related allergies was poor. Priority triggers like peanut and sesame showed conspicuously absent sensitization. This highlights the need for confirmatory testing and dietary counseling for patients in South India with perceived food-triggered allergic symptoms. Sensitization does not always imply clinical allergy, emphasizing personalized approaches and shared decision making. Avoiding tolerated foods solely on the basis of allergen relation is not recommended. Further research exploring component-resolved diagnosis and pollen allergy is needed. The dietary pattern in India may play a role in preventing food-related atopic diseases. Future studies with IgE inhibition assays could differentiate independent cosensitization and cross-reactivity, identifying primary sources of sensitization to specific allergens, proteins, and other components.

## Disclosure statement

Supported by 10.13039/501100001411ICMR, New Delhi, research grant 3/1/3/PDF(13)/2017-10.13039/100000171HRD.

Disclosure of potential conflict of interest: S. Nimmagadda is speaker for GlaxoSmithKlein and AstraZeneca. C. Venter reports grants from Reckitt Benckiser, 10.13039/100006423Food Allergy Research and Education, National Peanut Board; personal fees from Reckitt Benckiser, Nestlé Nutrition Institute, Danone, 10.13039/100011947Abbott Nutrition, Else Nutrition, Sifter, and Before Brands. R. Gupta reports research support from the NIH (R21 ID AI135705, R01 ID AI130348, U01 ID AI138907), FARE, Rho Inc, Melchiorre Family Foundation, Sunshine Charitable Foundation, Walder Foundation, UnitedHealth Group, 10.13039/100011033Thermo Fisher Scientific, Genentech, and the National Confectioners Association (NCA); serves as a medical consultant/advisor for 10.13039/100016185Aimmune Therapeutics, Before Brands, AllerGenis LLC, Kaléo Inc, Novartis, Genentech, DBV Technologies, and FARE; is employed by Ann & Robert H. Lurie Children’s Hospital of Chicago; and is professor of pediatrics and medicine at Northwestern University Feinberg School of Medicine. C. M. Warren reports research support from FARE and the Sunshine Charitable Foundation and is currently employed by 10.13039/100007059Northwestern University and 10.13039/100008250Northwestern University Feinberg School of Medicine. The rest of the authors declare that they have no relevant conflicts of interest.

## Supplementary data

eTables

Annexure I

Annexure II-a

Annexure II-b
