# Supplementary material for: Reported food-related symptoms and food allergen sensitization in a selected adult population in Hyderabad, India: A hospital-based survey
Source: J Allergy Clin Immunol Glob. 2023 Dec 23;3(2):100204. doi: 10.1016/j.jacig.2023.100204 (PMC10818074; doi:10.1016/j.jacig.2023.100204)
Supplement: eTables [file mmc1.docx]

**SUPPLEMENTARY MATERIAL**

**Table S01: List of food items (with their local names) reported by participants as being associated with diagnosed atopic disease(s) (N=100)- pilot study results**

| **Food items (N=77)** | | | | | |
| --- | --- | --- | --- | --- | --- |
| **Cereals & Pulses (14)** | **Vegetables (20)** | **Fruits (16)** | **Nuts (4)** | **Meat, fish, egg (7)** | **Other food items (15)** |
| Corn (Mokkajonna) | Beans (Beans) | Apple (Apple) | Almond (Badam) | Mutton (meka/gore mamsam) | Chocolate (Chocolate) |
| Jowar (Jonallu) | Bitter gourd (Kakarkaya) | Avocado (Venna pandu) | Cashew nut (Jeedi pappu) | Beef (aavu mamsam) | Black pepper (Miriyalu) |
| Rice (Biyyam) | Eggplant (Vankaya) | Banana (Aratipandu) | Pista (Pista) | Egg (Guddu) | Cardamom (Yalakulu) |
| Wheat (Godhuma) | Cabbage (Cabbage) | Chikoo (Sapota) | Walnut (Bikki pica) | Fish (chepalu) | Cinnamon (Dalchina chekka) |
| Black eyed bean (Bobbarlu) | Carrot (Carrot) | Grape (Draksa) |  | Prawn (Royyalu) | Cumin (Jeelakara) |
| Chickpea (Kabuli senagalu) | Cauliflower (Cauliflower) | Guava (Jamakaya) |  | Chicken (kodi mamsam) | Fennel seed (Sompu) |
| Green gram (Pesarlu) | Cucumber (Dosakaya) | Kiwi (Kiwi) |  | Pork (Pandi mamsam) | Sesame (Nuvvulu) |
| Green pea (Batani) | Drumstick (Munaga kada) | Lemon (Nimmakaya) |  |  | Honey (Thene) |
| Horse gram (Ulavalu) | Garlic (Velluli) | Mango (Mamidi pandu) |  |  | Curd (Perugu) |
| Red kidney bean (Chikkuduginjalu) | Ginger (Allam) | Muskmelon (Kharbuja) |  |  | Milk (Palu) |
| Soya bean (Soyabean) | Capsicum (Capsicum) | Orange (Narinja pandu) |  |  | Coffee (Coffee) |
| Split green gram (Pesarapappu) | Lady finger (Bendakaya) | Papaya (Boppayi) |  |  | Tea (Chai) |
| Split red gram (Kandi pappu) | Mushroom (Puttagodugu) | Peach (Peach) |  |  | Betel leaf (Tamala paku) |
| Split red lentil (Erra pappu) | Mustard leaves (Avaalu akalu) | Pineapple (Anasa pandu) |  |  | Arecanut (Vakka) |
|  | Onion (Ullipaya) | Strawberry (Strawberry) |  |  | Tamarind (Chinta pandu) |
|  | Potato (Aloogadda) | Watermelon (Pucchakay) |  |  | Coconut (Kobbari) |
|  | Pumpkin (Gummadikaya) |  |  |  |  |
|  | Radish (Mullangi) |  |  |  |  |
|  | Spinach (Palakura) |  |  |  |  |
|  | Tomato (Tomato) |  |  |  |  |

**Table S02: Agreement between perceived food related illness/disorder, positive skin prick tests and food specific IgE (N=1622)**

| **S. N** | **Food items** | **with perceived food related atopic disease(s)** | **SPT positive**  **(wheal ≥3mm diameter)** | **sIgE positive (>0.36 kUA/L)** |
| --- | --- | --- | --- | --- |
| **Cereals & Pulses** | | | | |
|  | Chickpea (Chingalu) | 524 (28.8) | 115 (6.3) | 21 (1.2) |
|  | Horse bean (Chenaga pappu) | 448 (24.6) | 0 (0) | 0 (0) |
|  | Wheat (Godhuma) | 298 (16.4) | 2 (0.1) | 0 (0) |
|  | Corn (Mokkajonna) | 286 (15.7) | 0 (0) | 0 (0) |
|  | Split red lentil (Erra pappu) | 260 (14.3) | 28 (1.5) | 0 (0) |
|  | Green pea (Batani) | 258 (14.2) | 0 (0) | 0 (0) |
|  | Jowar (Jonallu) | 246 (13.5) | 3 (0.2) | 0 (0) |
|  | Black eyed bean (Bobbarlu) | 238 (13.1) | 0 (0) | 0 (0) |
|  | Split red gram (Kandi pappu) | 234 (12.9) | 0 (0) | 0 (0) |
|  | Rice (Biyyam) | 232 (12.7) | 0 (0) | 0 (0) |
|  | Red kidney beans (Chikkuduginjalu) | 226 (12.4) | 0 (0) | 0 (0) |
|  | Green gram (Pesarlu) | 178 (9.8) | 0 (0) | 0 (0) |
|  | Soya bean (Soyabean) | 178 (9.8) | 0 (0) | 0 (0) |
|  | Split green gram (Pesarapappu) | 172 (9.5) | 0 (0) | 0 (0) |
| **Vegetables** | | | | |
|  | Cabbage (Cabbage) | 766 (42.1) | 39 (2.1) | 1 (0.1) |
|  | Drumstick (Mulakaya) | 518 (28.5) | 0 (0) | 0 (0) |
|  | Eggplant (Vankay) | 448 (24.6) | 27 (1.5) | 4 (0.2) |
|  | Garlic (Velluli) | 384 (21.1) | 14 (0.8) | 0 (0) |
|  | Capsicum (Capsicum) | 384 (21.1) | 0 (0) | 0 (0) |
|  | Ginger (Allam) | 306 (16.8) | 0 (0) | 0 (0) |
|  | Carrot (Carrot) | 302 (16.6) | 0 (0) | 0 (0) |
|  | Radish (Mullangi) | 302 (16.6) | 0 (0) | 0 (0) |
|  | Onion (Ullipaya) | 266 (14.6) | 0 (0) | 0 (0) |
|  | Mushroom (Puttagodugu) | 260 (14.3) | 0 (0) | 0 (0) |
|  | Mustard leaves (Avaalu) | 254 (14.0) | 0 (0) | 0 (0) |
|  | Cauliflower (Cauliflower) | 252 (13.8) | 10 (0.5) | 0 (0) |
|  | Tomato (tomato) | 232 (12.7) | 0 (0) | 0 (0) |
|  | Cucumber (Dosakay) | 230 (12.6) | 0 (0) | 0 (0) |
|  | Spinach (Palakura) | 224 (12.3) | 0 (0) | 0 (0) |
|  | Beans (Beans) | 178 (9.8) | 20 (1.1) | 0 (0) |
|  | Bitter gourd (Kakarkay) | 172 (9.5) | 0 (0) | 0 (0) |
|  | Lady finger (Bendakay) | 166 (9.1) | 0 (0) | 0 (0) |
|  | Pumpkin (Gummadikaya) | 166 (9.1) | 0 (0) | 0 (0) |
|  | Potato (Aloogadda) | 146 (8.0) | 0 (0) | 0 (0) |
| **Fruits** | | | | |
|  | Banana (Aratipandu) | 876 (48.1) | 0 (0) | 7 (0.4) |
|  | Grape (Draksa) | 870 (47.8) | 0 (0) | 0 (0) |
|  | Apple (Apil) | 766 (42.1) | 0 (0) | 0 (0) |
|  | Lemon (Nimmakaya) | 536 (29.5) | 0 (0) | 0 (0) |
|  | Orange (Narinja pandu) | 518 (28.5) | 0 (0) | 0 (0) |
|  | Strawberry (Strawberry) | 518 (28.5) | 3 (0.2) | 0 (0) |
|  | Mango (Mamidi pandu) | 396 (21.8) | 13 (0.7) | 0 (0) |
|  | Peach (Peach) | 350 (19.2) | 0 (0) | 0 (0) |
|  | Watermelon (Pucchakay) | 326 (17.9) | 0 (0) | 0 (0) |
|  | Avocado (Venna pandu) | 318 (17.5) | 0 (0) | 0 (0) |
|  | Papaya (Boppayi) | 306 (16.8) | 0 (0) | 0 (0) |
|  | Chikoo (Cheeko) | 242 (13.3) | 0 (0) | 0 (0) |
|  | Kiwi (Kiwi) | 234 (12.9) | 0 (0) | 0 (0) |
|  | Guava (Jamakaya) | 226 (12.4) | 0 (0) | 0 (0) |
|  | Pineapple (Anasa pandu) | 172 (9.5) | 0 (0) | 0 (0) |
|  | Muskmelon (Kharbuja) | 158 (8.7) | 0 (0) | 0 (0) |
| **Nuts** | | | | |
|  | Almonds (Badam) | 266 (14.6) | 0 (0) | 0 (0) |
|  | Cashew nut (Jidi pappu) | 266 (14.6) | 0 (0) | 0 (0) |
|  | Walnut (Bikki pica) | 224 (12.3) | 105 (5.8) | 20 (1.1) |
|  | Pista (Pista) | 220 (12.1) | 0 (0) | 0 (0) |
|  | **Non-veg food items** |  |  |  |
|  | Mutton (mutton) | 864 (47.5) | 16 (0.9) | 0 (0) |
|  | Beef (aavu mamsam) | 312 (17.1) | 0 (0) | 0 (0) |
|  | Egg (Guddu) | 280 (15.4) | 0 (0) | 0 (0) |
|  | Fish (cheyapalu) | 278 (15.3) | 0 (0) | 0 (0) |
|  | Prawn (Royyalu) | 246 (13.5) | 12 (0.7) | 0 (0) |
|  | Chicken (chicken) | 224 (12.3) | 0 (0) | 0 (0) |
|  | Pork (Pandi mamsam) | 178 (9.8) | 0 (0) | 0 (0) |
| **Other commonly consumed food items** | | | | |
|  | Curd (Perugu) | 778 (42.7) | 0 (0) | 0 (0) |
|  | Tea (Chai) | 448 (24.6) | 5 (0.3) | 0 (0) |
|  | Cinnamon (Dalchina chaka) | 350 (19.2) | 0 (0) | 0 (0) |
|  | Chocolate (Chocolate) | 338 (18.6) | 0 (0) | 0 (0) |
|  | Black pepper (Miriyalu) | 298 (16.4) | 0 (0) | 0 (0) |
|  | Milk (Palu) | 296 (16.3) | 0 (0) | 0 (0) |
|  | Arecanut (Vakka) | 286 (15.7) | 0 (0) | 0 (0) |
|  | Coffee (Coffee) | 286 (15.7) | 42 (2.3) | 0 (0) |
|  | Honey (Thene) | 266 (14.6) | 0 (0) | 0 (0) |
|  | Fennel seeds (Sompu) | 238 (13.1) | 0 (0) | 0 (0) |
|  | Cumin (Jeelakara) | 232 (12.7) | 107 (5.9) | 25 (1.4) |
|  | Cardamom (Elaichi) | 218 (12.0) | 0 (0) | 0 (0) |
|  | Sesame (Nuvvulu) | 172 (9.5) | 0 (0) | 0 (0) |
|  | Tamarind (Chinta pandu) | 172 (9.5) | 19 (1) | 0 (0) |
|  | Betel leaf (Tamala paku) | 152 (8.4) | 24 (1.3) | 2 (0.1) |
|  | Coconut (Kobbari) | 536 (29.5) | 28 (1.5) | 0 (0) |

**Table S03: Agreement between perceived food related illness, positive SPT and sIgE based on the age groups (N=1622)**

| **Adults 18-60 years (N=1622)** | | | | | | | | | | |
| --- | --- | --- | --- | --- | --- | --- | --- | --- | --- | --- |
|  | **Food items** | **Age group** | | | | | | | | |
|  |  | **18-25 years (n=233)** | | | **25-40 years (n=464)** | | | **40-60 years (n=925)** | | |
|  |  | with perceived food related atopic disease | SPT positive  (wheal ≥3mm diameter) | food sIgE positive (>0.36 kUA/L) | with perceived food related atopic disease | SPT positive  (wheal ≥3mm diameter) | food sIgE positive (>0.36 kUA/L) | with perceived food related atopic disease | SPT positive (wheal ≥3mm diameter) | food sIgE positive (>0.36 kUA/L) |
| **Cereals & Pulses** | | | | | | | | | | |
| 1 | Chickpea (Chingalu) | 53(22.7) | 7(3.0) | 3(1.3) | 124(26.7) | 42(9.1) | 8(1.7) | 314(33.9) | 60(6.5) | 8(0.9) |
| 2 | Horse bean (Chenaga pappu) | 58(24.9) | 0(0) | 0(0) | 132(28.4) | 0(0) | 0(0) | 185(20.0) | 0(0) | 0(0) |
| 3 | Wheat (Godhuma) | 30(12.9) | 0(0) | 0(0) | 60(12.9) | 0(0) | 0(0) | 181(19.6) | 2(0.2) | 0(0) |
| 4 | Corn (Mokkajonna) | 40(17.2) | 0(0) | 0(0) | 73(15.7) | 0(0) | 0(0) | 140(15.1) | 0(0) | 0(0) |
| 5 | Split red lentil (Erra pappu) | 25(10.7) | 2(0.9) | 0(0) | 55(11.9) | 13(2.8) | 0(0) | 108(11.7) | 12(1.3) | 0(0) |
| 6 | Green pea (Batani) | 24(10.3) | 0(0) | 0(0) | 68(14.7) | 0(0) | 0(0) | 137(14.8) | 0(0) | 0(0) |
| 7 | Jowar (Jonallu) | 24(10.3) | 0(0) | 0(0) | 67(14.4) | 2(0.4) | 0(0) | 126(13.6) | 1(0.1) | 0(0) |
| 8 | Black eyed bean (Bobbarlu) | 18(7.7) | 0(0) | 0(0) | 70(15.1) | 0(0) | 0(0) | 127(13.7) | 0(0) | 0(0) |
| 9 | Split red gram (Kandi pappu) | 35(15.0) | 0(0) | 0(0) | 64(13.8) | 0(0) | 0(0) | 126(13.6) | 0(0) | 0(0) |
| 10 | Rice (Biyyam) | 34(14.6) | 0(0) | 0(0) | 55(11.9) | 0(0) | 0(0) | 103(11.1) | 0(0) | 0(0) |
| 11 | Red kidney beans (Chikkuduginjalu) | 46(19.7) | 2(0.9) | 0(0) | 74(15.9) | 13(2.8) | 3(0.6) | 187(20.2) | 14(1.5) | 7(0.8) |
| 12 | Green gram (Pesarlu) | 29(12.4) | 0(0) | 0(0) | 50(10.8) | 0(0) | 0(0) | 77(8.3) | 0(0) | 0(0) |
| 13 | Soya bean (Soyabean) | 16(6.9) | 0(0) | 0(0) | 48(10.3) | 0(0) | 0(0) | 79(8.5) | 0(0) | 0(0) |
| 14 | Split green gram (Pesarapappu) | 35(15.0) | 0(0) | 0(0) | 43(9.3) | 0(0) | 0(0) | 89(9.6) | 0(0) | 0(0) |
| **Vegetables** | | | | | | | | | | |
| 15 | Cabbage (Cabbage) | 117(50.2) | 5(2.1) | 1(0.4) | 149(32.1) | 16(3.4) | 0(0) | 411(44.4) | 18(1.9) | 0(0) |
| 16 | Drumstick (Mulakaya) | 53(22.7) | 0(0) | 0(0) | 124(26.7) | 0(0) | 0(0) | 308(33.3) | 0(0) | 0(0) |
| 17 | Eggplant (Vankay) | 58(24.9) | 1(0.4) | 0(0) | 132(28.4) | 8(1.7) | 2(0.4) | 185(20.0) | 17(1.8) | 2(0.2) |
| 18 | Garlic (Velluli) | 72(30.9) | 2(0.9) | 0(0) | 79(17.0) | 1(0.2) | 0(0) | 196(21.2) | 6(0.6) | 0(0) |
| 19 | Capsicum (Capsicum) | 39(16.7) | 1(0.4) | 0(0) | 62(13.4) | 0(0) | 0(0) | 78(8.4) | 10(1.1) | 0(0) |
| 20 | Ginger (Allam) | 28(12.0) | 0(0) | 0(0) | 99(21.3) | 0(0) | 0(0) | 166(17.9) | 0(0) | 0(0) |
| 21 | Carrot (Carrot) | 48(20.6) | 0(0) | 0(0) | 72(15.5) | 0(0) | 0(0) | 139(15.0) | 0(0) | 0(0) |
| 22 | Radish (Mullangi) | 48(20.6) | 0(0) | 0(0) | 72(15.5) | 0(0) | 0(0) | 139(15.0) | 0(0) | 0(0) |
| 23 | Onion (Ullipaya) | 22(9.4) | 0(0) | 0(0) | 56(12.1) | 0(0) | 0(0) | 165(17.8) | 0(0) | 0(0) |
| 24 | Mushroom (Puttagodugu) | 22(9.4) | 0(0) | 0(0) | 52(11.2) | 0(0) | 0(0) | 163(17.6) | 0(0) | 0(0) |
| 25 | Mustard leaves (Avaalu) | 22(9.4) | 0(0) | 0(0) | 50(10.8) | 0(0) | 0(0) | 159(17.2) | 0(0) | 0(0) |
| 26 | Cauliflower (Cauliflower) | 24(10.3) | 0(0) | 0(0) | 67(14.4) | 3(0.6) | 0(0) | 132(14.3) | 7(0.8) | 0(0) |
| 27 | Tomato (tomato) | 29(12.4) | 0(0) | 0(0) | 61(13.1) | 0(0) | 0(0) | 102(11.0) | 0(0) | 0(0) |
| 28 | Cucumber (Dosakay) | 11(4.7) | 0(0) | 0(0) | 90(19.4) | 0(0) | 0(0) | 105(11.4) | 0(0) | 0(0) |
| 29 | Spinach (Palakura) | 11(4.7) | 0(0) | 0(0) | 82(17.7) | 0(0) | 0(0) | 101(10.9) | 0(0) | 0(0) |
| 30 | Beans (Beans) | 13(5.6) | 3(1.3) | 0(0) | 49(10.6) | 6(1.3) | 0(0) | 88(9.5) | 11(1.2) | 0(0) |
| 31 | Bitter gourd (Kakarkay) | 13(5.6) | 0(0) | 0(0) | 43(9.3) | 0(0) | 0(0) | 94(10.2) | 0(0) | 0(0) |
| 32 | Lady finger (Bendakay) | 29(12.4) | 0(0) | 0(0) | 44(9.5) | 0(0) | 0(0) | 71(7.7) | 0(0) | 0(0) |
| 33 | Pumpkin (Gummadikaya) | 16(6.9) | 0(0) | 0(0) | 48(10.3) | 0(0) | 0(0) | 73(7.9) | 0(0) | 0(0) |
| 34 | Potato (Aloogadda) | 9(3.9) | 0(0) | 0(0) | 33(7.1) | 0(0) | 0(0) | 70(7.6) | 0(0) | 0(0) |
| **Fruits** | | | | | | | | | | |
| 35 | Banana (Aratipandu) | 131(56.2) | 0(0) | 2(0.9) | 218(47.0) | 0(0) | 1(0.2) | 436(47.1) | 0(0) | 2(0.2) |
| 36 | Grape (Draksa) | 137(58.8) | 0(0) | 0(0) | 212(45.7) | 0(0) | 0(0) | 430(46.5) | 0(0) | 0(0) |
| 37 | Apple (Apil) | 117(50.2) | 0(0) | 0(0) | 149(32.1) | 0(0) | 0(0) | 411(44.4) | 0(0) | 0(0) |
| 38 | Lemon (Nimmakaya) | 39(16.7) | 0(0) | 0(0) | 154(33.2) | 0(0) | 0(0) | 279(30.2) | 0(0) | 0(0) |
| 39 | Orange (Narinja pandu) | 53(22.7) | 0(0) | 0(0) | 124(26.7) | 0(0) | 0(0) | 308(33.3) | 0(0) | 0(0) |
| 40 | Strawberry (Strawberry) | 33(14.2) | 2(0.9) | 0(0) | 154(33.2) | 0(0) | 0(0) | 267(28.9) | 1(0.1) | 0(0) |
| 41 | Mango (Mamidi pandu) | 72(30.9) | 1(0.4) | 0(0) | 90(19.4) | 8(1.7) | 0(0) | 197(21.3) | 4(0.4) | 0(0) |
| 42 | Peach (Peach) | 51(21.9) | 0(0) | 0(0) | 72(15.5) | 0(0) | 0(0) | 184(19.9) | 0(0) | 0(0) |
| 43 | Watermelon (Pucchakay) | 28(12.0) | 0(0) | 0(0) | 75(16.2) | 0(0) | 0(0) | 190(20.5) | 0(0) | 0(0) |
| 44 | Avocado (Venna pandu) | 28(12.0) | 0(0) | 0(0) | 105(22.6) | 0(0) | 0(0) | 166(17.9) | 0(0) | 0(0) |
| 45 | Papaya (Boppayi) | 28(12.0) | 0(0) | 0(0) | 99(21.3) | 0(0) | 0(0) | 166(17.9) | 0(0) | 0(0) |
| 46 | Chikoo (Cheeko) | 17(7.3) | 0(0) | 0(0) | 87(18.8) | 0(0) | 0(0) | 108(11.7) | 0(0) | 0(0) |
| 47 | Kiwi (Kiwi) | 35(15.0) | 0(0) | 0(0) | 55(11.9) | 0(0) | 0(0) | 114(12.3) | 0(0) | 0(0) |
| 48 | Guava (Jamakaya) | 39(16.7) | 0(0) | 0(0) | 62(13.40 | 0(0) | 0(0) | 84(9.1) | 0(0) | 0(0) |
| 49 | Pineapple (Anasa pandu) | 18(7.7) | 0(0) | 0(0) | 32(6.9) | 0(0) | 0(0) | 98(10.6) | 0(0) | 0(0) |
| 50 | Muskmelon (Kharbuja) | 9(3.9) | 0(0) | 0(0) | 33(7.1) | 0(0) | 0(0) | 82(8.9) | 0(0) | 0(0) |
| **Nuts** | | | | | | | | | | |
| 51 | Almond (Badam) | 25(10.7) | 0(0) | 0(0) | 69(14.9) | 0(0) | 0(0) | 127(13.7) | 0(0) | 0(0) |
| 52 | Cashew nut (Jidi pappu) | 37(15.9) | 0(0) | 0(0) | 72(15.5) | 0(0) | 0(0) | 136(14.7) | 0(0) | 0(0) |
| 53 | Walnut (Bikki pica) | 50(21.5) | 9(3.9) | 2(0.9) | 60(12.9) | 39(8.4) | 10(2.2) | 97(10.5) | 52(5.6) | 5(0.5) |
| 54 | Pista (Pista) | 39(16.7) | 0(0) | 0(0) | 62(13.4) | 0(0) | 0(0) | 78(8.4) | 0(0) | 0(0) |
| **Non-veg food items** | | | | | | | | | | |
| 55 | Mutton (mutton) | 131(56.2) | 1(0.4) | 0(0) | 212(45.7) | 5(1.1) | 0(0) | 430(46.5) | 9(1.0) | 0(0) |
| 56 | Beef (aavu mamsam) | 28(12.0) | 0(0) | 0(0) | 99(21.3) | 0(0) | 0(0) | 172(18.6) | 0(0) | 0(0) |
| 57 | Egg (Guddu) | 40(17.2) | 0(0) | 0(0) | 73(15.7) | 0(0) | 0(0) | 140(15.1) | 0(0) | 0(0) |
| 58 | Fish (cheyapalu) | 30(12.9) | 0(0) | 0(0) | 64(13.8) | 0(0) | 0(0) | 133(14.4) | 0(0) | 0(0) |
| 59 | Prawn (Royyalu) | 41(17.6) | 1(0.4) | 0(0) | 60(12.9) | 3(0.6) | 0(0) | 115(12.4) | 8(0.9) | 0(0) |
| 60 | Chicken (chicken) | 50(21.5) | 0(0) | 0(0) | 61(13.1) | 0(0) | 0(0) | 96(10.4) | 0(0) | 0(0) |
| 61 | Pork (Pandi mamsam) | 18(7.7) | 0(0) | 0(0) | 37(8.0) | 0(0) | 0(0) | 99(10.7) | 0(0) | 0(0) |
| **Other commonly consumed food items** | | | | | | | | | | |
| 62 | Curd (Perugu) | 117(50.2) | 0(0) | 0(0) | 160(34.5) | 0(0) | 0(0) | 412(44.5) | 0(0) | 0(0) |
| 63 | Tea (Chai) | 58(24.9) | 1(0.4) | 0(0) | 132(28.4) | 0(0) | 0(0) | 185(20.0) | 4(0.4) | 0(0) |
| 64 | Cinnamon (Dalchina chaka) | 46(19.7) | 0(0) | 0(0) | 77(16.6) | 0(0) | 0(0) | 184(19.9) | 0(0) | 0(0) |
| 65 | Chocolate (Chocolate) | 23(9.9) | 0(0) | 0(0) | 88(19.0) | 0(0) | 0(0) | 194(21.0) | 0(0) | 0(0) |
| 66 | Black pepper (Miriyalu) | 29(12.4) | 0(0) | 0(0) | 60(12.9) | 0(0) | 0(0) | 182(19.7) | 0(0) | 0(0) |
| 67 | Milk (Palu) | 37(15.9) | 0(0) | 0(0) | 77(16.6) | 0(0) | 0(0) | 150(16.2) | 0(0) | 0(0) |
| 68 | Arecanut (Vakka) | 46(19.7) | 0(0) | 0(0) | 73(15.7) | 0(0) | 0(0) | 140(15.1) | 0(0) | 0(0) |
| 69 | Coffee (Coffee) | 24(10.3) | 4(1.7) | 0(0) | 55(11.9) | 15(3.2) | 0(0) | 180(19.5) | 21(2.3) | 0(0) |
| 70 | Honey (Thene) | 37(15.9) | 0(0) | 0(0) | 66(14.2) | 0(0) | 0(0) | 136(14.7) | 0(0) | 0(0) |
| 71 | Fennel seeds (Sompu) | 35(15.0) | 0(0) | 0(0) | 57(12.3) | 0(0) | 0(0) | 106(11.5) | 0(0) | 0(0) |
| 72 | Cumin (Jeelakara) | 12(5.2) | 12(5.2) | 1(0.4) | 70(15.1) | 36(7.8) | 8(1.7) | 127(13.7) | 55(5.9) | 14(1.5) |
| 73 | Cardamom (Elaichi) | 50(21.5) | 0(0) | 0(0) | 55(11.9) | 0(0) | 0(0) | 96(10.4) | 0(0) | 0(0) |
| 74 | Sesame (Nuvvulu) | 16(6.9) | 0(0) | 0(0) | 54(11.6) | 0(0) | 0(0) | 73(7.9) | 0(0) | 0(0) |
| 75 | Tamarind (Chinta pandu) | 29(12.4) | 2(0.9) | 0(0) | 44(9.5) | 9(1.9) | 0(0) | 77(8.3) | 8(0.9) | 0(0) |
| 76 | Betel leaf (Tamala paku) | 9(3.9) | 2(0.9) | 0(0) | 38(8.2) | 9(1.9) | 0(0) | 71(7.7) | 12(1.3) | 2(0.2) |
| 77 | Coconut (Kobbari) | 33(14.2) | 3(1.3) | 0(0) | 156(33.6) | 9(1.9) | 0(0) | 278(30.1) | 14(1.5) | 0(0) |
