# Supplementary material for: Reported food-related symptoms and food allergen sensitization in a selected adult population in Hyderabad, India: A hospital-based survey
Source: J Allergy Clin Immunol Glob. 2023 Dec 23;3(2):100204. doi: 10.1016/j.jacig.2023.100204 (PMC10818074; doi:10.1016/j.jacig.2023.100204)
Supplement: Annexure I [file mmc2.docx]

**PILOT QUESTIONNAIRE**

**A HOSPITAL BASED PILOY SURVEY ON THE PREVALENCE OF FOOD ALLERGY IN HYDERABAD, INDIA**

**Name of the Hospital______________________________________________________**

**GENERAL INFORMATION**

Enrolment ID.: Date of enrolment:

Age (in years): Gender: Male/female

Contact no.:

**SOCIOECONOMIC STATUS**

**Education Occupation**

Professional degree & PG 7 Minister/Registrar/Chief/Official 12

Graduation 6 Professional/Management/Secretary 10

Intermediate 5 Semi-professional/PCS officer 6

High School 4 Clinical/shop owner/farm owner 5

Middle School 3 Semiskilled 3

Primary 1 literate 2 Unskilled 2

Illiterate 1 Unemployed 1

**Occupation code of father/husband or resource person (Dependent subject)**

**Income**

>10,000/- month 12 **SES Scale=I+II+III**

5000/- to 9,999/- 10 26-31 I Upper

4000/-to 4,999/- 6 16-25 II Upper Middle

2500/- to 3999/- 4 11-15 III Lower Middle

1500/- to 2499/- 3 5-10 IV Upper lower

501/- to 1499/- 2 <5 V Lower

<500/- month 1

**Diagnosis:**

- Asthma
- Allergic Rhinitis
- Atopic Dermatitis
- Urticaria
- GI induced allergic symptoms (for ex-allergic diarrhoea)

List food items associated (triggers/exacerbates) your diagnosis/illness:
