# Supplementary material for: Reported food-related symptoms and food allergen sensitization in a selected adult population in Hyderabad, India: A hospital-based survey
Source: J Allergy Clin Immunol Glob. 2023 Dec 23;3(2):100204. doi: 10.1016/j.jacig.2023.100204 (PMC10818074; doi:10.1016/j.jacig.2023.100204)
Supplement: Annexure II-a [file mmc3.docx]

**Section -I SCREENING QUESTIONNAIRE**

**A HOSPITAL BASED SURVEY ON THE PREVALENCE OF FOOD ALLERGY IN HYDERABAD, INDIA**

**Name of the Hospital______________________________________________________**

1. **GENERAL INFORMATION**

Enrolment no.: Date of registration:

Place of residence: Rural/ Urban

Age (in years): Gender: Male/female

Contact no.:

1. **SOCIOECONOMIC STATUS**

**Education Occupation**

Professional degree & PG 7 Minister/Registrar/Chief/Official 12

Graduation 6 Professional/Management/Secretary 10

Intermediate 5 Semi professional/PCS officer 6

High School 4 Clinical/shop owner/farm owner 5

Middle School 3 Semi Skilled 3

Primary 1 literate 2 Unskilled 2

Illiterate 1 Unemployed 1

**Occupation code of father/husband or resource person (Dependent subject)**

**Income**

>10,000/- month 12 **SES Scale=I+II+III**

5000/- to 9,999/- 10 26-31 I Upper

4000/-to 4,999/- 6 16-25 II Upper Middle

2500/- to 3999/- 4 11-15 III Lower Middle

1500/- to 2499/- 3 5-10 IV Upper lower

501/- to 1499/- 2 <5 V Lower

<500/- month 1

1. **HISTORY OF PRESENT ILLNESS**

**Diagnosis:**

- Asthma
- Allergic Rhinitis
- Atopic Dermatitis
- Urticaria
- GI induced allergic symptoms (for ex-allergic diarrhoea)

| **Have you ever had either of the above-mentioned disorder/illness caused by eating any of the following foods? Tick as many apply Food items (N=77)** | | | | | |
| --- | --- | --- | --- | --- | --- |
| **Cereals & Pulses (14)** | **Vegetables (20)** | **Fruits (16)** | **Nuts (4)** | **Non-veg (7)** | **Other food items (15)** |
| Corn (Mokkajonna) | Beans (Beans) | Apple (Apple) | Almonds (Badam) | Mutton (meka/gore mamsam) | Chocolate (Chocolate) |
| Jowar (Jonallu) | Bitter gourd (Kakarkaya) | Avocado (Venna pandu) | Cashew nut (Jeedi pappu) | Beef (aavu mamsam) | Black pepper (Miriyalu) |
| Rice (Biyyam) | Brinjal (Vankaya) | Banana (Aratipandu) | Pista (Pista) | Egg (Guddu) | Cardamom (Yalakulu) |
| Wheat (Godhuma) | Cabbage (Cabbage) | Chikoo (Sapota) | Walnut (Bikki pica) | Fish (chepalu) | Cinnamon (Dalchina chekka) |
| Black eyed bean (Bobbarlu) | Carrot (Carrot) | Grape (Draksa) |  | Prawn (Royyalu) | Cumin (Jeelakara) |
| Chickpea (Kabuli senagalu) | Cauliflower (Cauliflower) | Guava (Jamakaya) |  | Chicken (kodi mamsam) | Fennel seeds (Sompu) |
| Green gram (Pesarlu) | Cucumber (Dosakaya) | Kiwi (Kiwi) |  | Pork (Pandi mamsam) | Sesame (Nuvvulu) |
| Green pea (Batani) | Drumstick (Munaga kada) | Lemon (Nimmakaya) |  |  | Honey (Thene) |
| Horse gram (Ulavalu) | Garlic (Velluli) | Mango (Mamidi pandu) |  |  | Curd (Perugu) |
| Red kidney beans (Chikkuduginjalu) | Ginger (Allam) | Muskmelon (Kharbuja) |  |  | Milk (Palu) |
| Soya bean (Soyabean) | Capsicum (Capsicum) | Orange (Narinja pandu) |  |  | Coffee (Coffee) |
| Split green gram (Pesarapappu) | Lady finger (Bendakaya) | Papaya (Boppayi) |  |  | Tea (Chai) |
| Split red gram (Kandi pappu) | Mushroom (Puttagodugu) | Peach (Peach) |  |  | Betel leaf (Tamala paku) |
| Split red lentil (Erra pappu) | Mustard leaves (Avaalu akalu) | Pineapple (Anasa pandu) |  |  | Arecanut (Vakka) |
|  | Onion (Ullipaya) | Strawberry (Strawberry) |  |  | Tamarind (Chinta pandu) |
|  | Potato (Aloogadda) | Watermelon (Pucchakay) |  |  | Coconut (Kobbari) |
|  | Pumpkin (Gummadikaya) |  |  |  |  |
|  | Radish (Mullangi) |  |  |  |  |
|  | Spinach (Palakura) |  |  |  |  |
|  | Tomato (Tomato) |  |  |  |  |

1. **Total duration of illness:**

- Asthma
- Allergic Rhinitis
- Atopic Dermatitis
- Urticaria
- GI induced allergic symptoms (for ex-allergic diarrhoea)

1. **PHYSICAL ACTIVITY PATTERN**

- Sedentary/ Moderate/ Heavy
- Do you perform regular exercise? Yes/No

1. **DIET HISTORY**

- Dietary habit: Vegetarian/Non vegetarian/Eggarian
- How do you describe your apetite? Poor/Fair/Good
- Do you skip the meals? Yes/No
- Do you prefer snacks in between the meals? Yes/No; If yes, give details
  - Fried snacks like patties, pakoda, chips.
  - Roasted snacks like chana, moong, peanut, bhelpuri
  - Fruit salad, green salad
- Food likes
- Food dislikes

1. **FOOD ALLERGY RELATED INFORMATION**

- Any F/H of food allergy- Yes/ No; Provide details:
- Total no. Of hours you sleep: Sound sleep/disturbed sleep
- Do you go for work/school/daily activities regularly: Yes/No?
- Did your reaction come on quickly, usually within an hour after eating the food: Yes/No? Provide details:
- Did allergy medicine help? For example-Antihistamines should relieve hives- Yes/ No
- Is your reaction always associated with a certain food? Yes/ No
- Did anyone else in the house who ate the same food get sick? Yes/ No; Provide details-
- How much did you eat before you had a reaction?
- How was the food prepared? (Provide details for every food item perceived as associated to the illness)
- Did you eat other food at the same time you had the reaction? Yes/ No; Provide details-

**If patient is eligible for the main study, will be continued otherwise will be provided dietary counselling.**
