# Supplementary material for: Reported food-related symptoms and food allergen sensitization in a selected adult population in Hyderabad, India: A hospital-based survey
Source: J Allergy Clin Immunol Glob. 2023 Dec 23;3(2):100204. doi: 10.1016/j.jacig.2023.100204 (PMC10818074; doi:10.1016/j.jacig.2023.100204)
Supplement: Annexure II-b [file mmc4.docx]

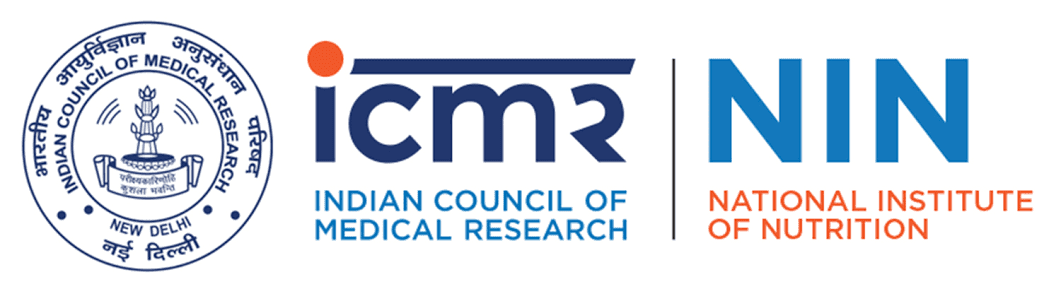


**NIN-Food Allergy and Allergens in India (NIN-FAAI)**

*Hyderabad Questionnaire*

Instructions for Interviewers:

- 1. During the meeting, greet the participant politely and explain the purpose.
  2. Explain the PIS and ICF in detail.
  3. Obtain the consent, complete the interview, and thank the participant.
  4. Please use your soft skills to the best of your ability to convince and collect required information from the participant.
  5. Please try to get the questionnaire completed within 20-30 minutes.

| Date |  | Place |  |
| --- | --- | --- | --- |
| Name of Interviewer |  | | |
| Name of the Hospital & Department |  | | |
| Name of the Collaborator |  | | |
| Other relevant information |  | | |

| Diagnosis:   - Allergic Rhinitis - Asthma - Urticaria - Atopic Dermatitis - Allergic Diarrhoea |
| --- |

| Personal Information | |
| --- | --- |
| 1. Name & ID number: |  |
| 2. Date of Birth |  |
| 3. Contact Address & Ph no. |  |

| Demographic Information | | | | | | | |
| --- | --- | --- | --- | --- | --- | --- | --- |
| 4. Gender | Male | Female | 5. Age | Months | 6. Height & Weight |  | |
| 7. Ethnicity  Country of Origin, If not India |  | | | 8. Place of Birth (City, State, Country) |  | | |
| 9. What town/city you currently live in? |  | | | 10. Have you lived in this town/city since birth? | | | **Y/N** |

| 11. Socio-economic status and other factors. (Refer Kuppuswamy Scale -Annexure I) | |
| --- | --- |
| 12. Family history of any disease | Y/N, if yes specify |
| 13. Environmental settings | Rural/Urban |

| 14. Did this food related illness or problem include:(from screening Qare)  14.1 Itching and urticaria  14.2 Breathlessness  14.3 Runny stuffy nose  14.4 Diarrhea or vomiting (not food poisoning)  14.5 Headache  14.6 Fainting or dizziness | | | Y/N  Y/N  Y/N  Y/N  Y/N  Y/N | | |
| --- | --- | --- | --- | --- | --- |
| 15. Duration of symptoms   - 1. 1-5 years   2. 5-10 years   3. 10-15 years   4. 15-20 years   5. 20-25 years   15.6 >25 years | | | Y/N  Y/N  Y/N  Y/N  Y/N  Y/N | | |
| 16. When you are near animals (such as cats, dogs, or horses etc.) and/or trees, grass, or flowers, or when there is a lot of pollen, do you ever  16.1 start to cough?  16.2 start to wheeze?  16.3 get a feeling of tightness in your chest?  16.4 start to feel short of breath?  16.5 get a runny or stuffy nose or start to sneeze?  16.6 get itchy or watering eyes? | | | | Y/N  Y/N  Y/N  Y/N  Y/N  Y/N | |
| 17. Which time of year does this happen?  17.1 winter  17.2 spring  17.3 summer  17.4 autumn | | | | Y/N  Y/N  Y/N  Y/N | |
| 18. Smoking  18.1 Smoking history  18.2 Smoke inhalation  18.3 Tobacco Intake | Ex-smoker/Current smoker/No smoking  Active/Passive/None  Y/N | | | | |
| 19. Laboratory investigations   - 1. Serum IgE   19.2 SPT against reported food items as allergens (to be attached)  19.3 Food Specific Ig E for reported food items as allergens (to be attached/tabulated)  20. Any other relevant information?  21. Are you willing to be contacted again for further food allergy studies? | | | Y/N  Y/N | | |

###### **Thank you very much for completing this questionnaire!**
